# Computational modeling of PET tracer distribution in solid tumors integrating microvasculature

**DOI:** 10.1186/s12896-021-00725-3

**Published:** 2021-11-25

**Authors:** Niloofar Fasaeiyan, M. Soltani, Farshad Moradi Kashkooli, Erfan Taatizadeh, Arman Rahmim

**Affiliations:** 1 Department of Mechanical Engineering, K. N. Toosi University of Technology, Tehran, Tehran Province Iran; 2 Department of Civil Engineering, Polytechnique University, Montreal, QC Canada; 3 Department of Electrical and Computer Engineering, University of Waterloo, Waterloo, ON Canada; 4 Centre for Biotechnology and Bioengineering (CBB), University of Waterloo, Waterloo, ON Canada; 5 Advanced Bioengineering Initiative Center, Computational Medicine Center, K. N. Toosi University of Technology, Tehran, Tehran Province Iran; 6 School of Biomedical Engineering, University of British Columbia, Vancouver, BC Canada; 7 Departments of Radiology and Physics, University of British Columbia, Vancouver, BC Canada; 8 Department of Integrative Oncology, BC Cancer Research Institute, Vancouver, BC Canada

**Keywords:** Solid tumor, Positron Emission Tomography (PET), Microvascular network, FDG radiotracer, Convection–Diffusion-Reaction modeling

## Abstract

**Background:**

We present computational modeling of positron emission tomography radiotracer uptake with consideration of blood flow and interstitial fluid flow, performing spatiotemporally-coupled modeling of uptake and integrating the microvasculature. In our mathematical modeling, the uptake of fluorodeoxyglucose F-18 (FDG) was simulated based on the Convection–Diffusion–Reaction equation given its high accuracy and reliability in modeling of transport phenomena. In the proposed model, blood flow and interstitial flow are solved simultaneously to calculate interstitial pressure and velocity distribution inside cancer and normal tissues. As a result, the spatiotemporal distribution of the FDG tracer is calculated based on velocity and pressure distributions in both kinds of tissues.

**Results:**

Interstitial pressure has maximum value in the tumor region compared to surrounding tissue. In addition, interstitial fluid velocity is extremely low in the entire computational domain indicating that convection can be neglected without effecting results noticeably. Furthermore, our results illustrate that the total concentration of FDG in the tumor region is an order of magnitude larger than in surrounding normal tissue, due to lack of functional lymphatic drainage system and also highly-permeable microvessels in tumors. The magnitude of the free tracer and metabolized (phosphorylated) radiotracer concentrations followed very different trends over the entire time period, regardless of tissue type (tumor vs. normal).

**Conclusion:**

Our spatiotemporally-coupled modeling provides helpful tools towards improved understanding and quantification of in vivo preclinical and clinical studies.

**Supplementary Information:**

The online version contains supplementary material available at 10.1186/s12896-021-00725-3.

## Background

Cancer is a major cause of death worldwide, and it is estimated that the number of people diagnosed with cancer will increase in the coming decades [1]. Positron emmission tomography (PET) is a powerful imaging modality towards improved diagnosis, prognosis, staging, restaging and treatment response monitoring of cancer patients [2, 3]. The entirety of phenomena underlying radiotracer uptake in PET imaging still explored. Mathematical modeling of biological systems is a powerful scheme, towards improved understanding and quantification, towards design of more effective clinical trials [4–7].

To simulate radiotracer phenomena, correlation of the tissue time activity curve (TAC) to the underlying tumor physiology has been used [8]. Conventional compartment models (including Patlak plots) focus on analysis of temporal uptake without coupling uptake spatially [9–15]; by contrast it is possible to employ transport modeling, as we have pursued out elsewhere [16, 17], to provide complete spatiotemporal coupling. Solute transport modeling has been used widely to simulate drug delivery working based on the Convection–Diffusion-Reaction (CDR) equation. Several studies have been done in this field before [18–23]. This approach is based on using partial differential equations (PDEs) in contrast to the compartmental modeling methods which are based on ordinary differential equations (ODEs).

Conventional compartmental modeling assumes that there are separate pools of tracer concentrations called compartments [24]. By contrast, the CDR equation leads to investigate spatiotemporal changes of drug or radiotracer uptake which cannot be achieved by the compartmental modeling method. This method considers the effects of various parameters such as the interstitial velocity and pressure, capillary network structure, and permeability of the tissues on PET tracer distribution which are not integrated within compartmental modeling methods. In spatiotemporal-based modeling, the different effects of convection, diffusion, reaction, and binding to cells can be incorporated [17, 25]. As an example, some three-dimensional (3D) simulations were developed to study drugs transport in a peritoneal tumor during the intraperitoneal chemotherapy and effects of tumor geometries and sizes, vascular normalization therapy, drug diffusivity, necrotic core, and tissue permeability on the drug delivery [26, 27]. We pursued out a similar approach to simulate tracer [17] and drug delivery mechanisms [28] governed by tumor transport phenomena. None of the above-mentioned studies consider the structure of microvasculature, and either simplified homogenous tracer/drug release in entire tumor domain or in some cases one-dimensional (1D) synthetic capillary networks were employed. As tumor microvasculature provides nutrients, oxygen, and glucose for the tumor growth [29], its effect on solute transport is inevitable, so it should be considered in the geometry. Additionally, the size and density of capillaries vary in different tissues, so using the synthetic capillary structure can result in unrealistic outcomes compared to in vivo studies.

A number of studies have been conducted on the formation of capillaries around and within tumors. These studies have used mathematical modeling to generate tumor microvasculature [30]. In our past effort [17], which was based on previously employed microvascular networks produced by Anderson et al. [31], we generated a continuous two-dimensional (2D) capillary network and used a reinforced random walk to follow the movement of endothelial cells [25, 30, 32, 33]. No past efforts have used geometry of capillary networks which is taken from a synthetic image for simulation purposes, and only computational-based capillary generations have been used. In our recent studies, the CDR equation was used to investigate the Fluoromisonidazole (FMISO) tracer [34] and targeted drug delivery [35] distribution in the solid tumor. However, as current medical instruments cannot detect nano- and micro-scale sized microvasculature in tissues due to their poor accuracy, inserting the image of the capillaries into the numerical simulation for further implementations has remained a significant challenge. To tackle this problem, several studies have been proposed to detect the microvasculature structure from medical images. As clinical images contain background noise, image processing techniques must be carried out for detection of capillaries. Numerous filters have been introduced to cancel out the noise effect including single-scale and multi-scale matched filters [36–39], single-scale and multi-scale Gabor filters [40, 41], and Bar-selective Combination of Shifted Filter Response [42]. In the latter study, a new filter was proposed, combining previous filters in a novel way. This filter has a direction-independent ability to detect any bar-like structure, making it a good candidate for detection of microvasculature.

None of the previously mentioned studies carried out mathematical modeling of tracer delivery via structures of capillaries as extracted from medical images. To fill this gap, the present study aims to examine FDG PET imaging through computational approach. The concentration of FDG tracer was calculated based on combination of 5 K-compartmental method with CDR equations. Our study couples both time and space as key factors in FDG tracer distribution in both normal and tumor regions. To solve for interstitial pressure, velocity, and concentration of FDG, intravascular flow inside capillaries and interstitial fluid flow inside tissues are coupled. Subsequently, the distribution of the FDG tracer is calculated. As such, we aim to enhance quantification and assessment of uptake in individual patients and tissues. Next, we elaborate our methodology, followed by results, discussion, and conclusion.

## Results and discussion

### Pressure and velocity distribution

**Fig. 1:**
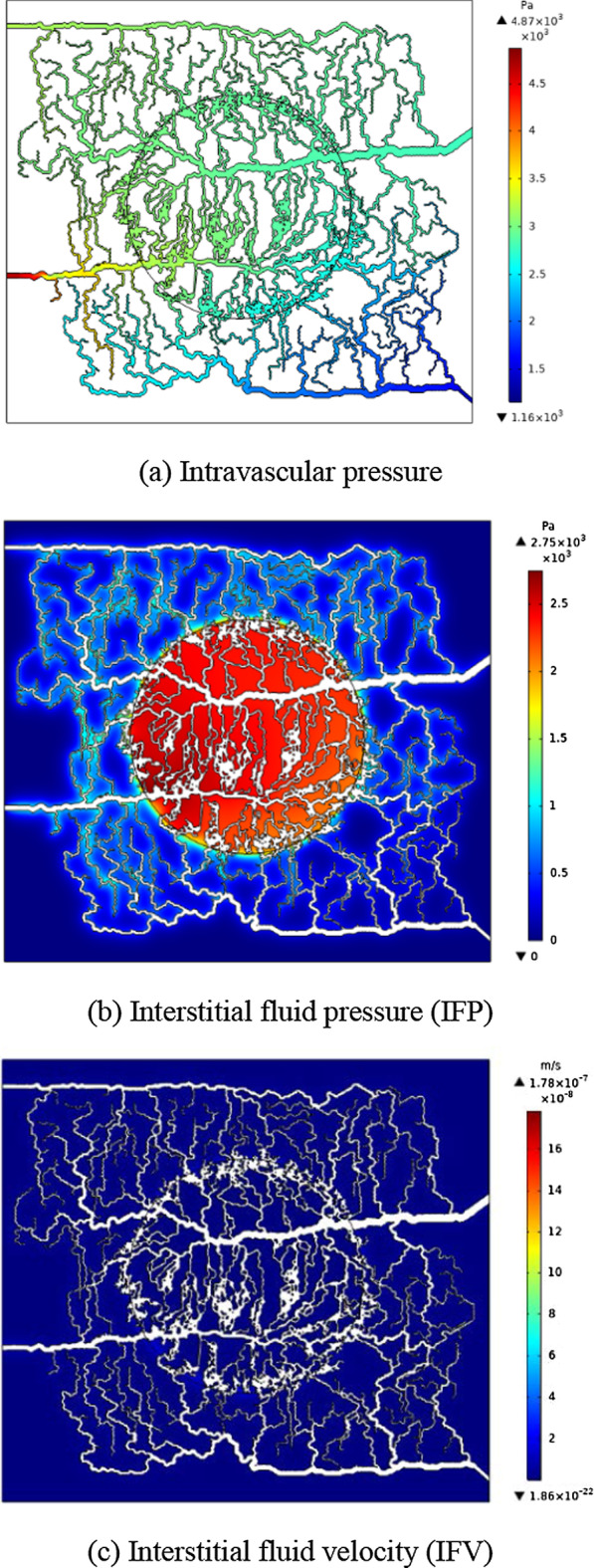
Distribution of blood pressure, IFP, and IFV: **a** intravascular (blood) pressure, **b** IFP within normal and tumor tissues, **c** IFV in both normal and tumor tissues

The obtained intravascular pressure and interstitial fluid pressure distributions are shown in Fig. 1. The maximum value of the interstitial fluid pressure (IFP) is 2.74 kPa in the tumor region due to the leaky behavior of the capillaries in solid tumor regions along with the lack of lymphatic vessels in tumor compared to normal tissue. Additionally, IFP is higher in the area where capillaries are closer together, in both normal and tumor tissues, *i.e.*, the IFP is proportional to the microvascular density. The heterogeneous capillary network as source terms in interstitial fluid flow equation cause heterogeneous IFP distribution in both tumor and normal tissues. Interstitial fluid velocity (IFV) can be obtained in the whole tissue domain as there is a direct relation between IFP and IFV in the Darcy’s law. As can be seen from Fig. 1c, the value of the IFV is extremely low (maximum value ~ 1.78e−7 m/s & median value ~ 4.5e−8 m/s).

### FDG tracer distribution

**Fig. 2:**
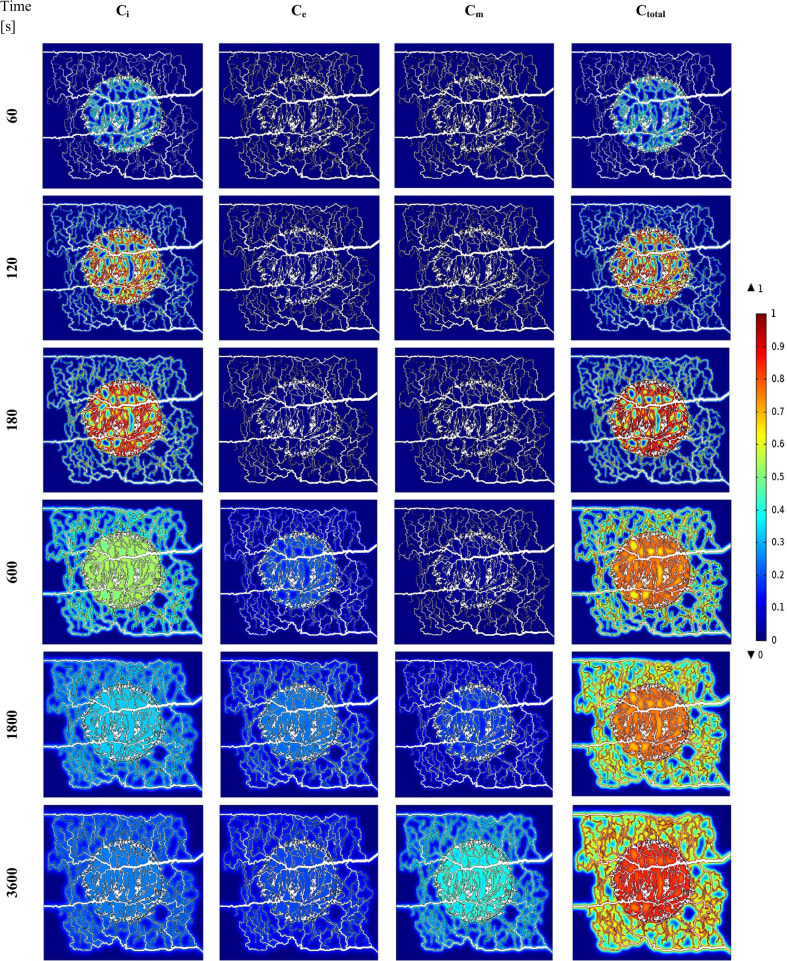
Spatiotemporal distribution of FDG radiotracer including extracellular (column 1), intracellular (column 2), phosphorylated intracellular (column 3), and total (column 4) concentrations, shown at 6 different time frames. Different concentrations are normalized to maximum value of total concentration. The same color bar is used for all plots

Spatiotemporal distribution of FDG radiotracer concentrations (including C_i_; extracellular, C_e_; intracellular, C_m_; phosphorylated intracellular, and C_total_; total concentrations) are demonstrated in Fig. 2. To provide the most distinguishable vision for all of the concentrations, different concentrations are normalized to maximum value of total concentration (C_m_) at six different time frames. It is seen that at the very beginning of tracer infusion, free tracer concentration (C_i_) is dominant compared to both intracellular concentration (C_e_) and phosphorylated (metabolized) intracellular concentration (C_m_). With passage of time, C_e_ and C_m_ increase, first the former, followed by the latter. At the beginning, C_i_ dominates the total concentration, but as time passes, the amount of C_i_ is reduced, and C_e_ and C_m_ dominate the total concentration.

As it is observed, C_i_ and C_e_ follow different trends from one another. Furthermore, the total FDG tracer concentration in tumor region is significantly higher than in surrounding normal tissue region at all time frames. This happens because of the great rate of extravasation from the capillary network in the tumor region and higher microvascular density (MVD) in this area. Maximum total concentration takes place in the tumor area, and is multiple times greater compared to concentration in the surrounding normal tissue.

Next, we calculated the average value of the total concentration (in time and space) separately in tumor and normal tissues. Results show that average value of C_total_ in tumor region is higher than the average value of C_total_ in normal tissue. As seen in Fig. 2, regions with higher MVD, have higher concentration compared to regions with less dense capillaries or none. It should be mentioned that for each time step, the maximum value is different. The location where the maximum value of concentration occurs is nearly at the center of the tumor region. For more clarification, the median value of different concentrations at each time points within or outside of the tumor are reported and compared in Table 1.

**Table 1:** The median value of different concentrations at each time points within or outside of the tumor

Time [s]	Tissue type	C_i_ (mol/m^3^)	C_e_ (mol/m^3^)	C_m_(mol/m^3^)	C_total_(mol/m^3^)
60	Tumor	23.1867	0.2301	1051.0168	1074.4336
	Normal	2.0102	0.01781	108.3105	110.3385
120	Tumor	106.2470	2.10696	2587.4268	2695.7808
	Normal	12.0157	0.2142	338.3387	350.5686
180	Tumor	235.5401	7.51638	3040.0453	3283.1018
	Normal	32.3107	0.9013	532.7309	565.9429
600	Tumor	856.0214	140.0836	2243.2288	3239.3339
	Normal	216.5706	29.0337	778.7145	1024.3189
1800	Tumor	1056.0064	798.8236	1412.9760	3267.8060
	Normal	430.3229	254.8346	686.42605	1371.5835
3600	Tumor	829.3591	1689.3651	1055.1704	3573.8946
	Normal	416.8322	667.4632	570.2119	1654.5072

**Fig. 3:**
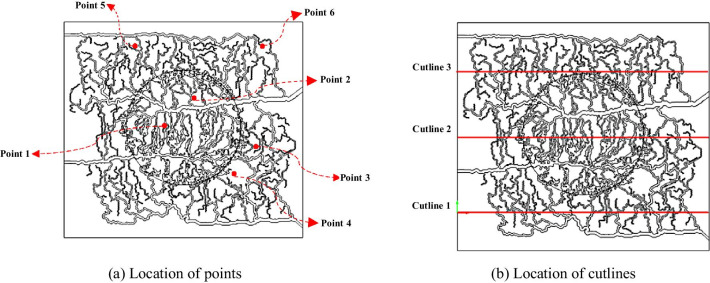
The location of points and cutlines which were used to calculate the average compartmental concentration values of FDG tracer

Time–space-averaged values of the extracellular tracer concentration (C_i_), intracellular concentration (C_e_), and phosphorylated intracellular concentration (C_m_) of FDG tracer were calculated. The aforementioned concentrations were averaged along three cutlines and at six points, as shown in Fig. 3. These points were chosen at locations with different physical properties: point #1 (in the area with high MVD) and #2 (in the area with low MVD) are located in tumor region, point #3 (in the area with high MVD) and #4 (in the area with low MVD) are in the normal tissue region (near the tumor region), and point #5 (in the area with high MVD) and #6 (in the area with low MVD) are positioned in the normal tissue region where they are far from the solid tumor. With the same approach, the locations of cutlines were chosen.

**Fig. 4:**
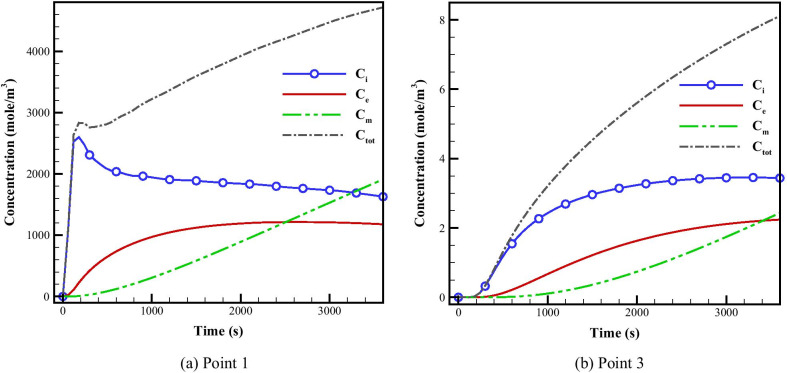
The averaged FDG tracer compartmental concentration distribution versus time for point 1

**Fig. 5:**
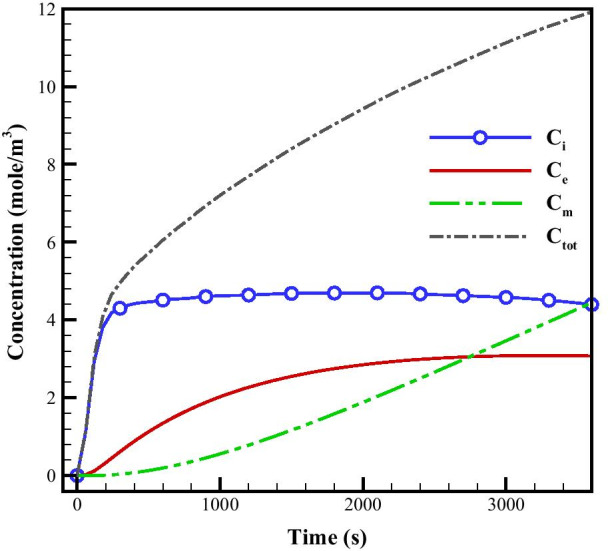
The temporal evolution of FDG tracer uptake in different compartments along cutline 1

As it can be seen in Fig. 3, we have selected different spots in various areas of domain to comparison the effect of microvascular density on IFP and tracer concentration. In the areas where the microvascular density is higher, the IFP is also higher. For instance, IFP for point#1 is about 2.7 kPa and for point#2 which is located in an area with lower microvascular density is about 2.55 kPa. The quantitative results for concentrations are shown in Figs. 4 and 5 and also Additional file 1: Figs. S2 and S3. The average concentrations at point #1 (Fig. 4a) is higher than the other five points as this point is located in tumor region with a high MVD. As it can be seen in Fig. 4 and Additional file 1: Fig. S2, the maximum total concentration for this point (point #1) is about 5000 mol/m^3^ at 3600 s, but for the other points, especially those which located in normal tissue, such as point #4 with the maximum total concentration below 1 mol/m^3^, this quantity is much lower. Although point #2 (Additional file 1: Fig. S2a) is also located within the tumor region, it has lower concentration (maximum total concentration ~ 4000 mol/m^3^) in comparison to point #1. This is due to the high impact of MVD on the concentration of FDG tracer. The same behavior is observed by comparing points #3 (Fig. 4b) vs. #4 (Additional file 1: Fig. S2b) and also point #5 (Additional file 1: Fig. S2c) vs. #6 (Additional file 1: Fig. S2d) to consider the effect of microvascular network’s structure on tracer distribution. Results demonstrate that FDG tracer concentration are higher at point #3 compared to point #4 due to the higher MVD. As it is shown in Figs. 4 and Additional file 1: Fig. S2, the maximum total concentration for point #3 is 8 mol/m^3^ which is about eight times higher than the maximum total concentration for point #4 (1 mol/m^3^). By observing point #5 and #6, the effect of microvasculature is also clearly visible. Point #5 is located in a region with a higher MVD in comparison to point #6, so the concentration at point #5 is higher than point #6. In any case, points #1 and #2 still have higher concentration values because of their location inside the solid tumor region. The reason for these results is the high permeability of the capillaries in the tumor compared to the normal tissue. Additionally, metabolism rate in cancer tissue is higher than in normal tissue.

Cutline #2 passing through the tumor region with the maximum total concentration of 1400 mol/m^3^ depicts the highest FDG tracer concentration (Additional file 1: Fig. S3a) compared to the other two cutlines. However, cutline #1 (Fig. 5) passes through the normal tissue region with a low MVD and it is located far from the solid tumor in comparison to cutline #3 (Additional file 1: Fig. S2b), and thus has the lowest concentration value (~ 12 mol/m^3^).

At early stages (0–600 [s]), FDG tracer activity (Figs. 4 and 5 and Figs. S2 and S3) follows the same trend as the plasma concentration profile (i.e., C_P_ profile). At that period in time, change in free tracer concentration (C_i_) is dominant compared to other compartmental concentrations. Intracellular tracer concentration (C_e_) has significant values at final stages (1800-3600 s).

The tumor microenvironment is very complex to be accurately represented by a single mathematical model. Therefore, the modeling used in the present study contains a number of assumptions. One of the most important assumptions is using 2D model instead of 3D model. Based on the literature [43], the effect of 3D modeling on spatial-mean parameters such as internalized drug is insignificant in the prostate tumor. A 2D model is also used in other studies including Jain and co-authors [44, 45], Soltani et al. [17, 34, 46], Chou et al. [47], and Stephanou et al. [30]. The next assumption is that the uniform transport characteristics, as well as uniform tumor cell density are considered regardless of the intra-or inter-tumor heterogeneity. The blood flow, in most cases, is laminar, even in the aorta. Considering blood flow in capillary as laminar flow should be fine, but the impact of red blood cell on the blood flow should be investigated in future studies, as the capillary dimension in some locations is comparable to the diameter of red blood cells. In addition, blood flow in capillaries should be considered as two-phase flow because it includes cells and plasma. Another parameter not accounted in this study is the rheology of blood. The static microvascular network extracted from an image is considered in this study, while the dynamic structure of capillary network should be investigated to consider the effects of shear stress, hemodynamic stimuli, and metabolic stimuli in the modeling. In general, due to the limitations and the lack of experimental verification, the predictions of the model described in the current study should be considered as qualitative instead of quantitative.

In future efforts, our main focus would be on using real extracted images from individual patients coupled with 3D modeling. In addition, we will utilize advanced tumor growth modeling and heterogeneous shapes of tumors to study tracer distributions. Another area of ongoing work is the inverse problem of estimating parameters of interest (such as diffusion) from imaging data.

### Validation of numerical model results

Numerous studies [6, 18, 23, 48] have addressed high IFP as the most significant obstacle to effective drug delivery to solid tumors. The results for IFP in present study have good agreement with the experimental results of Huber et al. [49] and Arfin et al. [50]; and also numerical study of Moradi Kashkooli et al. [51] and Sefidgar et al. [25]. The non-uniform distribution of IFV in tumor region has also been reported in Zhao et al. [52] and Pishko et al. [53]. Although the IFV values in current study are not equal to observations of Hompland et al. [54], their order of magnitude is the same. The obtained result for IFV has also been reported in the experimental work of Butler et al. [55] and also in the numerical studies of Pishko et al. [53], Sefidgar et al. [25], and Moradi Kashkooli et al. [35, 51]. Besides, the FDG tracer uptake value agrees well with the experimental results published by Carson [56] and Sha [57] research groups.

**Fig. 6:**
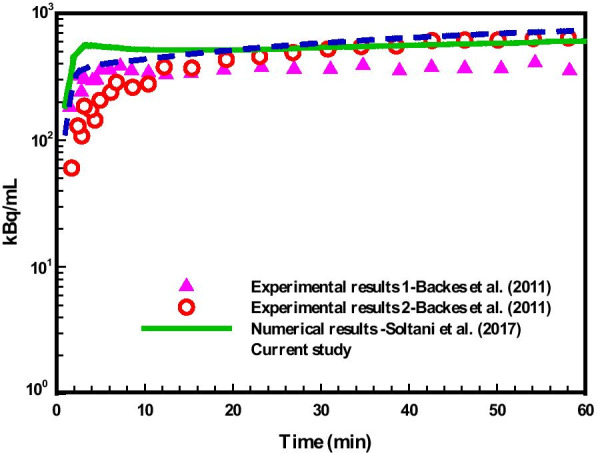
Comparison of the results of current study with two experimental results [21] and numerical results [17]

Figure 6 compares FDG concentration in tumor area against (*i*) experimental observation of Backes et al. [21] in which two different methods were used to estimate tracer kinetic constants (Table 2) and (*ii*) numerical results of Soltani et al. [17] which simulated the distribution of FDG tracer concentration in a synthetic mathematical-derived capillary network based on CDR equation. Total uptake in both extracellular and intracellular spaces is calculated, as measured in radionuclide imaging. Because the domain and conditions of experimental and modeling are different, the results do not exactly match. However, the total uptake of FDG in tumor for numerical simulation demonstrate nearly similar trend to experimental data. It is observed in the study that, after 15 min, the quantity of total concentration is very close to total concentration in the experimental results, and after 20 min the results exactly match with experimental data-2.

**Table 2:** Summary of parameters used in solute transport modeling

Parameter	Symbol [unit]	Value	References
Effective diffusion coefficient	$D_{eff}$[mm^2^/s]	0.37e−9 (Normal Tissue) 2.5e−3 (Tumor Tissue)	[70]
Coefficient of microvascular’s permeability	P [m/s]	3.75E−7 (Normal) 3.00E−6 (Tumor)	[43, 47]
Coefficient of filtration reflection	$\sigma_{f}$	0.9	[25]
Constant transport rate	*L*_3_ [1/min]	8.2e−4 (Normal Tissue)	[17]
Constant transport rate	*L*_4_ [1/min]	6.7e−4 (Normal Tissue)	[17]
Constant transport rate	*L*_5_ [1/min]	5.3e−4	[17]

## Conclusion

In the present work, we aimed to integrate realistic microvasculature structure within mathematical modeling of PET FDG tracer distribution using the CDR equation. The employed image processing method in this work enables consideration of any 2D synthetic or real microvasculature structure which could be expanded to 3D images for modeling the tracer distribution. The effects of intravascular and extravascular fluids were investigated by coupling the blood and interstitial flows. The results of interstitial pressure and blood pressure are consistent with experimental data. Maximum interstitial pressure occurred in solid tumor compared to normal tissue due to the lack of lymphatic drainage system along with the leaky nature of capillaries in the solid tumor. Subsequently, FDG uptake patterns were investigated by coupling previously calculated interstitial pressure and velocity with the CDR equation. Results demonstrated that FDG tracer concentration decreases by increasing the distance from capillaries due to the low rate of FDG tracer diffusion coefficient. In the regions with high microvascular density, the dependence of FDG distribution on capillary network can be seen clearly. Our framework leads to comprehensive mathematical modeling of tracer distribution in tissues for each image of interest.

Computational models cannot consider all complexities of the real world, though our models are being continually expanded. Notably, the Warburg effect is an important hypothesis of FDG PET imaging. The glucose metabolic rate can depend on several physiological factors of tumor microenvironment such as hypoxia, glucose transporter (GLUT), hexokinase (HK), and acidity [58]. These effects will be considered in future works. Additionally, it might be more realistic to extract vessels from histological slides or vascular networks from the dorsal skinfold window chamber model.

Given the higher resolution of our method compared to PET images, to compare simulation results with PET images data, an approach would be reduce the accuracy of our outputs through mapping methods (so that for example multiple pixels of our simulation would correspond to one pixel of PET); this can for instance be performed through careful simulation of the imaging process (forward modeling, resolution and noise degradation, followed by inverse problem). In the case of applying capillary-based transport simulation to validate or explain PET imaging, future efforts include enhancement of simulation studies with biopsy/pathology samples, on which the microvasculature can be imaged and compared with the PET scan prior to biopsy. Another direction in future work is to tackle the inverse problem for estimating parameters of interest taken from imaging data. This also includes decoding structure of the microvascular network from a tracer distribution of PET image data through a combination of inverse methods and multi-objective optimization, rather than directly solving the tracer distribution on a real/synthetic microvasculature by solute transport models.

## Methods

### Generation of microvasculature structure by image processing techniques

**Fig. 7:**
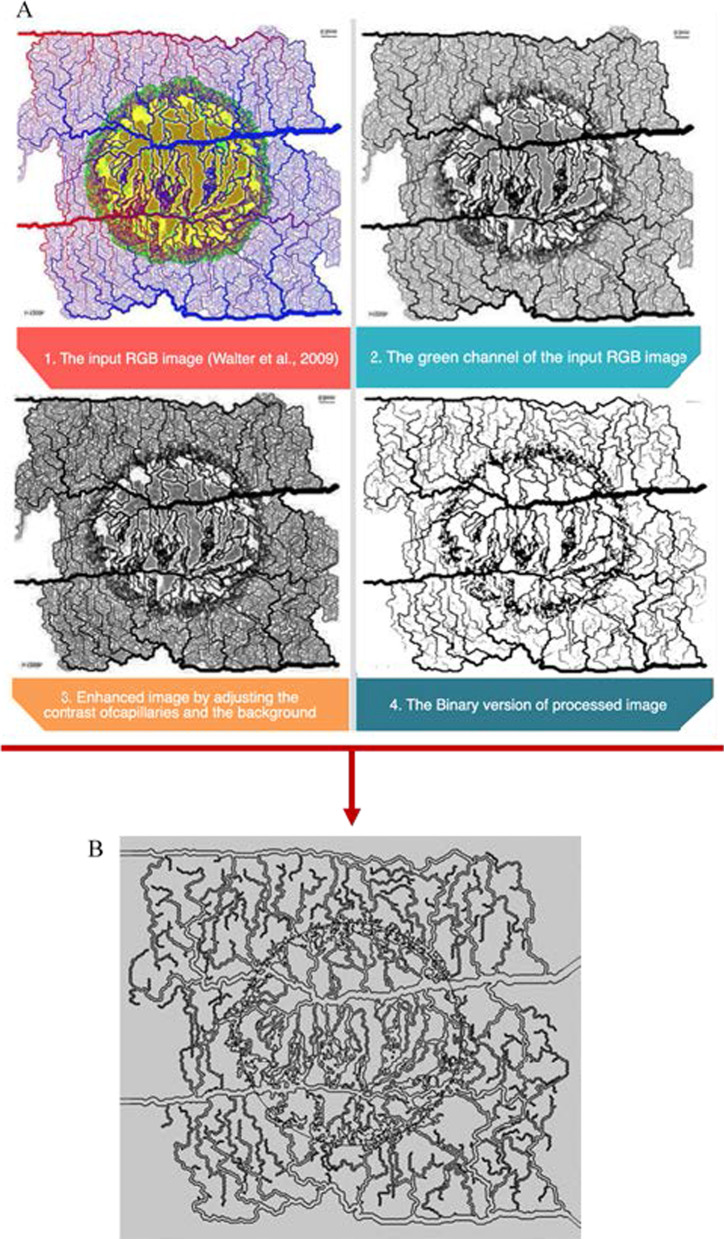
The steps were followed to improve quality of the input color image **a** and make it as the computational domain **b** for FEM analysis. Reproduced with permission from [59]

To generate the two-dimensional computational domain from the image consisting of capillaries, the contrast and quality of the image must be improved. In general, input images have background noise, low contrast, and homogeneous colors. As a result, image processing techniques must be taken into account before extracting the microvasculature regions. Additionally, due to the lack of availability of high-resolution clinical data (around micrometer or 100 nm), an image from Welter et al. [59] is used for this step. To generate the 2D computational domain from the image consisting of capillaries, the contrast and quality of the image must be improved. In general, input images have background noise, low contrast, and blurred uptake. As a result, image processing techniques must be considered before extracting the microvasculature regions. Additionally, due to a lack of availability of high-resolution clinical data (around micrometer or 100 nm), an image from Welter et al. [43] is used for this step. The steps taken are shown in Fig. 7. First, a green channel of the red–green–blue (RGB) image was extracted as it has the highest contrast compared to red and blue channels [60], and vessels and the background can be distinguished more easily. Next, the contrast-limited adaptive histogram equalization algorithm [61] was used to enhance the contrast of the green channel of the image by minimizing a variation in color intensities. These steps make it possible to recognize the capillaries areas from the background more effectively compared to the raw RGB image. After contrast improvement, a binary image was created from the pre-processing output, which means that each pixel with a higher value than the specified threshold is assigned a 1 value; otherwise 0. The threshold value is chosen in a trial-and-error manner which is different for every image. This step should be performed because the binary version of the image shows where noise patterns are more dense and in order to remove noise prior to the next step. Based on the subsequent image, minimum contour values were calculated in which the maximum accuracy belongs to the border of the capillaries. In the last step, the image contour value was calculated to find the boundaries of capillary walls. These boundaries determine edges of the closed surface which was then imported as the computational domain for finite element method (FEM) analysis. In the last step, the image contour value was calculated to find the boundaries of capillaries’ wall. These boundaries determine edges of the closed surface which was then imported as the computational domain for FEM analysis. These steps are shown in Fig. 7.

### Governing equations for solute transport modeling

To simulate FDG tracer uptake and distribution in the body tissues, the governing CDR equation must be fully coupled with both fluid flow inside the capillaries and interstitial flow in the tissues.

In CDR modeling, different steps for the tracer delivery to tumor region must be followed such as transportation of the tracer from vascular region to interstitial space. The transport of tracer is divided into three main parts: tracer transport in extracellular space by diffusion and convection mechanisms, internalized into the cells, and then intracellular tracer phosphorylation [62–64]. Combining the solute transport CDR equation with the standard 5 K compartmental model for FDG tracer in porous media, including consideration of source/sink terms [48, 65–67], we arrive at:

1 $$\frac{{\partial C_{i} }}{\partial t} = \nabla \cdot \left[ {D_{eff} \nabla C_{i} } \right] - v_{i} \nabla \cdot \left( {C_{i} } \right) - L_{3} C_{i} + L_{4} C_{e} + \Phi_{bt} - \Phi_{Lt}$$

2 $$\frac{{\partial C_{e} }}{\partial t} = L_{3} C_{i} - \left( {L_{4} + L_{5} } \right)C_{e}$$

3 $$\frac{{\partial C_{m} }}{\partial t} = L_{5} C_{e}$$

where $C_{i}$: Extracellular tracer concentration; $C_{e}$: Intracellular tracer concentration; $C_{m}$: Phosphorylated intracellular concentration (FDG-6-P); $D_{eff}$: Tracer effective diffusion coefficient; $v_{i}$: Interstitial flow velocity; $\Phi_{bt}$: Tracer transport rate in unit of volume from blood vessels to interstitial space; $\Phi_{Lt}$: Tracer transport rate in unit of volume from interstitial space to lymphatic drainage system; $L_{3}$ and $L_{4}$: Transport rates; and $L_{5}$: Phosphorylation rate.

In these equations, $\Phi_{{{\text{bt}}}}$ and $\Phi_{{{\text{Lt}}}}$ are respectively the tracer exchange rate per unit volume through the blood microvessels into the extracellular matrix (ECM), and from the ECM into the lymphatic drainage system. $\Phi_{{{\text{bt}}}}$ is defined based on Patlak’s model, as follows [48, 68, 69]:

4 $$\Phi_{bt} = \phi_{b} (1 - \sigma_{f} )C_{p} + \frac{PS}{V}(C_{p} - C_{i} )\frac{Pe}{{e^{Pe} - 1}}$$

5 $$Pe = \frac{{\phi_{b} (1 - \sigma_{f} )}}{{P\frac{S}{V}}}$$

in which *Pe* is the Peclet number, illustrating the convection rate to the diffusion rate, *σ*_*f*_ is the coefficient of filtration reflection, *P* is the coefficient of capillary permeability, *S/V* is the surface area per unit volume, *C*_*i*_ is extracellular tracer concentration, and $C_{P}$ is the tracer concentration in the inlet of microvessels.

The lymph term is considered to be distributed uniformly, only in normal tissue. The rate of tracer transport via lymphatic drainage system has been assumed to be as follow [48, 68, 69]:

6 $$\Phi_{Lt} = \phi_{L} C_{i}$$

**Fig. 8:**
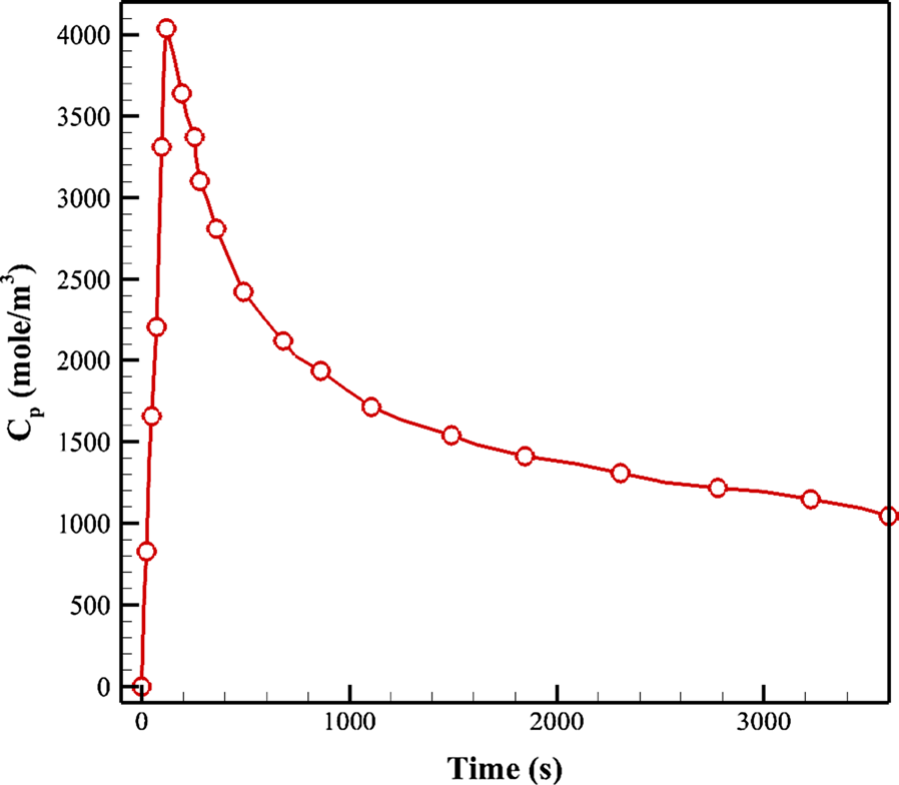
The variation of plasma arterial concentration of FDG tracer in blood (*C*_*P*_) versus time

The material properties of FDG tracer are shown in Table 2. As the tracer concentration varies by time, the concentration profile of *C*_*P*_, plasma arterial concentration of FDG tracer in blood, was used according to the previous study of Backes et al. [21], as is shown in Fig. 8.

To calculate compartmental concentration, first $v_{i}$ must be derived and then be implemented in Eq. (1). To simulate the interstitial fluid flow, the continuity equation in the porous media (here tissue regions) is modified to include the source ($\phi_{b}$) and sink ($\phi_{L}$) terms [18, 32, 61, 71–73]:

7 $$\nabla \cdot v_{i} = \phi_{b} - \phi_{L}$$

The source term is defined based on the differences of the intravascular fluid pressure from IFP and the intravascular osmotic pressure from interstitial osmotic pressure which is defined as follows [51, 72]:

8 $$\begin{aligned} \phi_{b} & = L_{p} \left( \frac{S}{V} \right)(P_{b} - P_{i} - \sigma_{s} (\pi_{b} - \pi {}_{i})) \\ \phi_{L} & = L_{PL} \left( \frac{S}{V} \right)_{L} (P_{i} - P_{L} ) \\ \end{aligned}$$

where $L_{P}$: Vascular hydraulic conductivity; $\frac{S}{V}$: Surface area per unit volume; $P_{b}$: Blood pressure; $P_{i}$: Interstitial fluid pressure; $\sigma_{s}$: Average osmotic reflection coefficient; $\pi_{b}$: Blood osmotic pressure; $\pi_{i}$: Interstitial fluid osmotic pressure, *L*_pL_(*S/V*)_L_: Lymphatic filtration coefficient; and P_L_: Hydrostatic pressure of lymphatic vessels.

In order to find the value of $P_{b}$ and $P_{i}$, the laminar flow and Darcy’s law must be solved in intravascular and interstitial regions, respectively.

The combination of Eq. (7) with Darcy’s law leads to:

9 $$- \kappa \nabla^{2} P_{i} = \phi_{b} - \phi_{L}$$

where $\kappa$ is interstitial hydraulic conductivity.

The employed material properties in the laminar flow and Darcy’s law are listed in Table 3.

**Table 3:** The material properties of the tumor and normal tissues [6, 17]

Parameter	Symbol [unit]	Value
Plasma osmotic pressure	$\pi_{b}$[mmHg]	20
Interstitial fluid osmotic pressure	$\pi_{i}$[mmHg]	10 (Normal tissue) 15 (Tumor tissue)
Average osmotic reflection coefficient	$\sigma_{s}$[−]	0.91 (Normal tissue) 0.82 (Tumor tissue)
Hydraulic conductivity of the microvascular wall	$L_{p}$[cm/((mmHg) s)]	0.36e−7 (Normal tissue) 2.8e−7 (Tumor tissue)
Interstitial hydraulic conductivity	$\kappa$[m^2^/(Pa s)]	6.41e−15 (Normal tissue) 30.0e−15 (Tumor tissue)
Lymphatic filtration coefficient	*L*_pL_(*S/V*)_L_ [1/(mmHg s)]	1.33e−5 (Normal tissue) 0 (Tumor tissue)
Hydrostatic pressure of lymphatic vessels	P_L_ [Pa]	0

### Computational domain, grid independency and boundary conditions

The computational domain includes a rectangle (6.72 cm × 6.09 cm) representing the normal tissue along with a tumor region which is shown as circle (d_tumor_ = 2.3 cm) located at the center. Four parent vessels are located at up and down sides and the middle of the rectangle which are connected to microvasculature network.

**Fig. 9:**
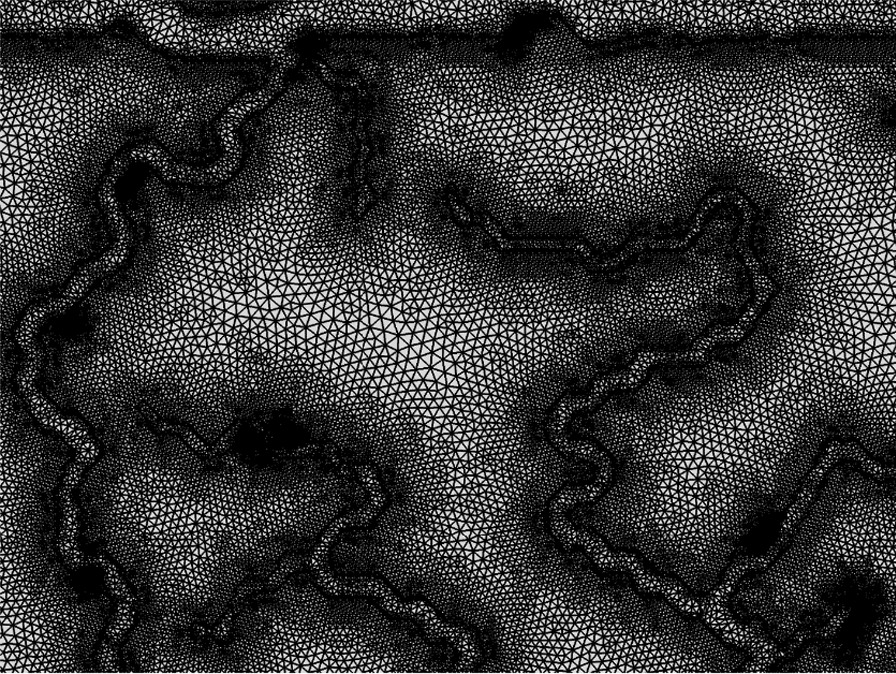
A zoomed-in view of the mesh element structure

Grid independency examination is carried out to demonstrate the effect of variation of mesh elements number on the simulation results. Suitable number of elements is selected by a trade-off between the computational cost and the accuracy of numerical results. When the finer mesh does not vary the results significantly, this mesh is assumed as an appropriate mesh. To this aim, various computational mesh—coarse, normal, fine, finer, and extra fine—are generated. With a fine mesh (5 times the primary mesh numbers), less than 2.5% change in concentration of FDG tracer and fluid flow parameters was observed. By enhancing the mesh numbers to finer and extra fine, which have respectively 10 and 20 times the primary mesh numbers, almost no changes in the concentration of FDG tracer and fluid flow parameters is found. As a consequence, the fine mesh elements (with the element number of 2887540) are employed in this study because of its lowest computational costs compared to other ones. It should be noted that triangular mesh type is utilized in the current study for tumor and normal tissue as shown in Fig. 9.

Between tumor and normal regions, the continuity boundary condition was assumed which consisted of the concentration, and concentration flux as follows:

10 $$\left( {D_{eff}^{t} \nabla C + v_{i} C} \right)\left| {\Omega^{ - } } \right. = \left( {D_{eff}^{n} \nabla C + v_{i} C} \right)\left| {\Omega^{ + } } \right.$$

11 $$C\left| {\Omega^{ - } } \right. = C\left| {\Omega^{ + } } \right.$$

In the above equations, $\Omega^{ - }$ and $\Omega^{ + }$ indicate tumor and normal tissue regions, repectively. Furthermore, an open boundary condition was considered for all four edges of the rectangle to prevent accumulation of the interstitial fluid in the domain and impliment the mass transfer across boundaries including convective inflow and outflow. The open boundary condition is expressed as follows.

12 $$- n \cdot \nabla C = 0$$

**Fig. 10:**
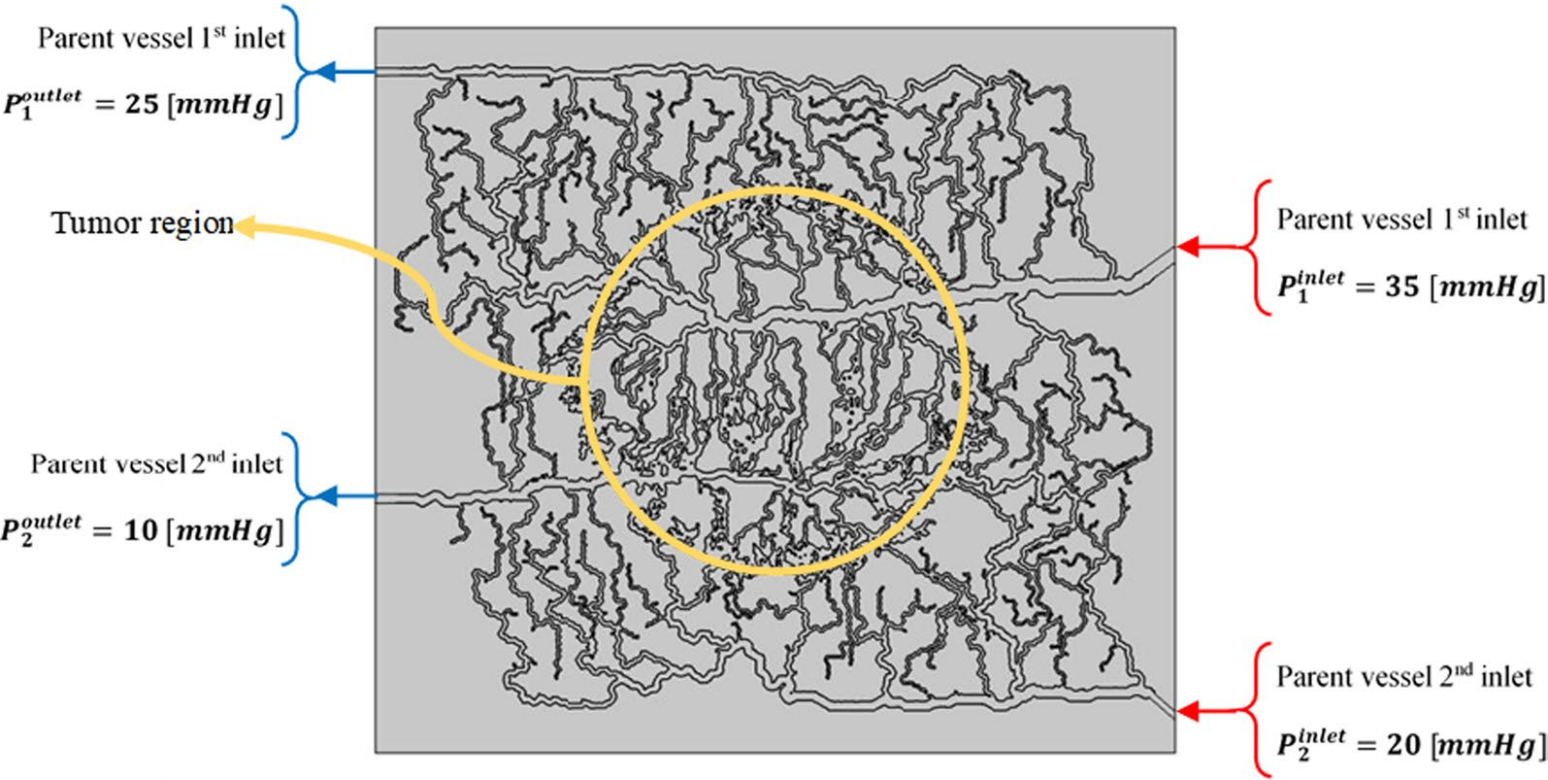
Computational domain and boundary conditions for intravascular flow

Computational domain considering tumor, normal tissue, and microvascular network along with the boundary conditions are shown in Fig. 10.

### Numerical solution details

There exist two phases of the solution for this study: steady-state and time-dependent one. Laminar intravascular flow, IFV, and IFP are obtained in steady-state phase. In time-dependent phase, Using the information of the previous step, tracer concentrations are achieved in tumor and normal tissue. First, geometry of tumor and its vascular network are extracted from image-processing of a synthetic tumor. Then, the mass and momentum equations in the vascular network and interstitial space are solved using an iterative approach. The resulting IFP and IFV values are then utilized to solve the CDR equations. Subsequently, the temporary CDR equations are solved to obtain different concentrations. COMSOL Multiphysics software is used for meshing the geometry. Moreover, all the governing equations -including continuity, Darcy, and CDR equations—are also solved by the commercial CFD software COMSOL Multiphysics 5.5 (COMSOL Inc, Stockholm, Sweden) based on the FEM which works by continuous Galerkin approach. Additionally, the residual square errors are set to 4 orders of magnitudes. We have different domains in our simulation space: vascular network and interstitial space which are connected to each other via transvascular exchange, as demonstrated in Additional file 1: Fig. S1.

## Supplementary Information

**Additional file 1:** Different domains in our simulation as well as the temporal FDG tracer concentration for different points and cutlines.

## Data Availability

The data supporting the conclusions of this article are included within the article.

## Abbreviations

PET: Positron emission tomography
FDG: Fluorodeoxyglucose F-18
CDR: Convection–Diffusion–Reaction
1D: One-dimensional
3D: Three-dimensional
IFP: Interstitial fluid pressure
MVD: Microvascular density
HK: Hexokinase
FEM: Finite element method
ECM: Extracellular matrix
TAC: Time activity curve
PDE: Partial differential equation
ODE: Ordinary differential equation
2D: Two-dimensional
FMISO: Fluoromisonidazole
IFV: Interstitial fluid velocity
GLUT: Glucose transporter
RGB: Red–green–blue
FDG-6-P: Phosphorylated intracellular concentration
