# Supplementary material for: Computational modeling of PET tracer distribution in solid tumors integrating microvasculature
Source: BMC Biotechnol. 2021 Nov 25;21:67. doi: 10.1186/s12896-021-00725-3 (PMC8620574; doi:10.1186/s12896-021-00725-3)
Supplement: Supplementary file 1 — Additional file 1: Different domains in our simulation as well as the temporal FDG tracer concentration for different points and cutlines. [file 12896_2021_725_MOESM1_ESM.docx]

**Supplementary Information**

**Computational modeling of FDG PET tracer distribution in solid tumors integrating microvasculature**

Niloofar Fasaeiyan, M. Soltan, Farshad M. Kashkooli, Erfan Taatizadeh, Arman Rahmim

| 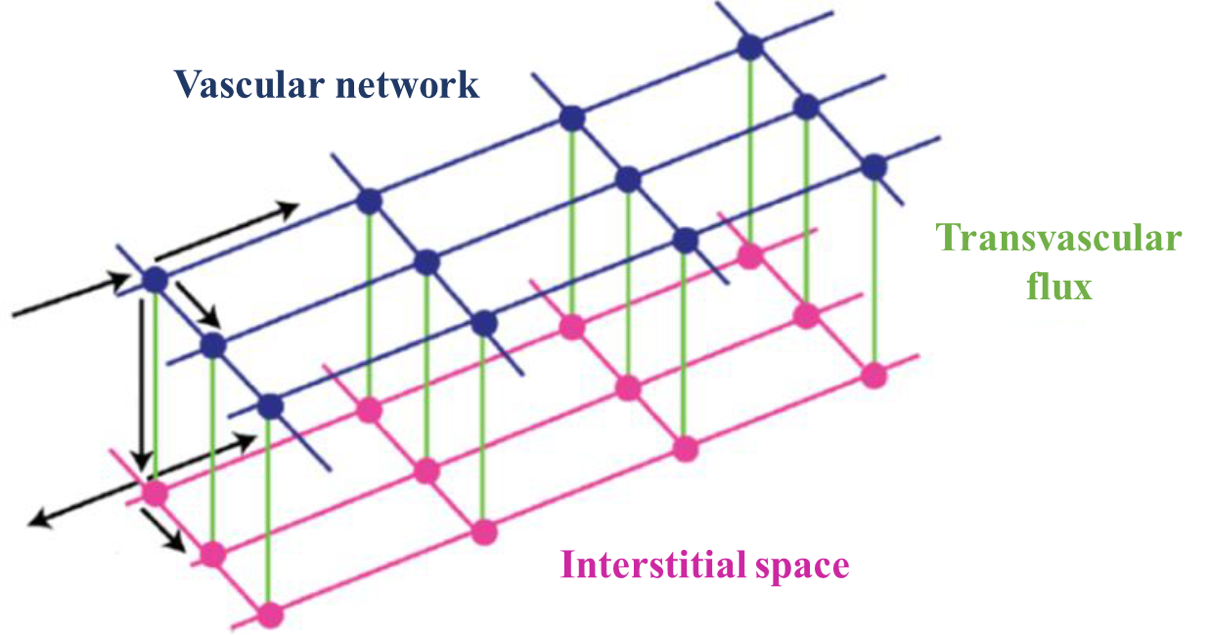 |
| --- |
| **Fig. S1.** Different domains in our simulation space including vascular network and interstitial space which are connected to each other via transvascular exchange. |

| **** |  |
| --- | --- |
| (a) Point 2 | (b) Point 4 |
|  |  |
| (c) Point 5 | (d) Point 6 |
| **Figure S2.** The averaged FDG tracer compartmental concentration distribution versus time for different points. | |

|  |  |
| --- | --- |
| (a) Cutline 2 | (b) Cutline 3 |
| **Figure S3.** The temporal evolution of FDG tracer uptake in different compartments along different cutlines. | |
